# “Day 25”: a temporal indicator of stabilization of mortality risk among COVID-19 patients with high viral load

**DOI:** 10.1186/s41182-022-00483-8

**Published:** 2022-12-09

**Authors:** Nancy A. Osman, Mona H. Hashish, Wafaa M. K. Bakr, Nermin A. Osman, Eman A. Omran

**Affiliations:** 1 Ministry of Health and Population, Alexandria, Egypt; 2 Department of Microbiology, High Institute of Public Health, Alexandria University, 165 El-Horreya Avenue, El-Ibrahimia, Alexandria, Egypt; 3 Biomedical Informatics and Medical Statistics Department, Medical Research Institute, Alexandria University, Alexandria, Egypt

**Keywords:** Cycle threshold, Cumulative mortality, COVID-19, CO-RAD, Predictors of mortality

## Abstract

**Introduction:**

The relationship between SARS-CoV-2 viral load and hospitalization and mortality among COVID-19 patients has been established. However, the estimation of the duration of time after which the risk of mortality of these patients stops escalating was not extensively discussed earlier. Stratifying patients according to their risk of mortality would optimize healthcare services and costs and reduce mortality.

**Methodology:**

In this retrospective observational study**,** hospital records were used to collect data of 519 COVID-19 patients from May through November 2020. Data included the clinical condition of patients, their viral loads, their admission chest computed tomography results (CO-RAD scale), and the duration of their hospitalization. A Kaplan–Meier analysis was constructed to estimate mortality risk concerning viral load.

**Results:**

By the end of the study, 20.42% of patients were deceased. The cumulative mortality was: 36.1% (75/208) among patients with high viral load, 12.6% (28/222) in those with moderate viral load, and 3.4% (3/89) among those with low viral load. Predictors of mortality were: older age [adjusted hazard ratio (aHR) = 1.02, 95% CI: [1.00–1.03], (*p* = 0.05)], "being female" [aHR = 1.53 with 95% CI: [1.03–2.26], (*p* = 0.031), "high CO-RAD scale" [aHR = 1.32 (1.06–1.64), *p* = 0.013], "high viral load" [aHR = 4.59 (2.38–20.92), *p* = 0.017, ICU admission [aHR = 15.95; 95%CI:7.22–35.20, *p* < 0.001] and lymphocytosis [aHR = 1.89 45;95%CI:1.04–3.45, *p* = 0.036]. In the ICU-admitted patients, the median survival was 19 days and mortality stabilized at "day 25". For patients with high viral load, mortality rates stabilized at "day 25 post-admission" after which the risks of mortality did not change until day 40, while patients with low and moderate viral loads reached the peak and stabilized at day "20 post-admission".

**Conclusions:**

Initial high SARS-CoV-2 viral load might be used as an indicator of a delayed stabilization of mortality risk among COVID-19 patients.

**Supplementary Information:**

The online version contains supplementary material available at 10.1186/s41182-022-00483-8.

## Introduction

The global pandemic caused by the “Severe Acute Respiratory Syndrome Coronavirus 2” (SARS-CoV-2) had an unprecedented effect on the rise of global mortality rates. According to the World Health Organization (WHO), the global excess mortality associated with Coronavirus Disease 2019 (COVID-19) was 14.91 million in the 24 months between 1 January 2020 and 31 December 2021 [1]. The mortality rate among Egyptian hospitalized patients with COVID-19 was estimated to reach 6.7% [2], which is notably higher than in other countries in the Eastern Mediterranean and Gulf Regions [3, 4]. Several factors might contribute to this variation; including differences in the criteria of hospitalization as well as patient-specific characteristics.

Clinical signs of COVID-19 are variable and include loss of taste and smell, fever, dry cough, gastrointestinal symptoms, and pneumonia. Common laboratory findings in COVID-19 patients include leucopenia (and often leucocytosis), elevated serum D-dimer, ferritin, and C-reactive protein (CRP) levels. The typical findings in chest computed topographies (CT) of COVID-19 patients are patchy, rounded, segmental, and sub-segmental ground glass opacities that may lead to consolidation [5]. The COVID-19 Reporting and Data System (CO-RAD) is a categorical assessment scheme for chest CT in patients suspected of having COVID-19, representing the level of suspicion for pulmonary involvement. Developed by the Dutch Radiological Society, this scale ranges from 1 (very low) to 5 (very high degree of suspicion), while the scale “6” refers to polymerase chain reaction (PCR)-confirmed cases [6].

The diagnosis of COVID-19 depends on the reverse transcriptase–quantitative polymerase chain reaction (RT-qPCR), and the results are generally reported as positive or negative. PCR provides also an indirect measure of the viral load in the sample, reflected by the value of the “cycle threshold” (Ct), which represents the number of amplification cycles required for the target gene to exceed a threshold level. Ct values are therefore inversely related to viral load and can provide an indirect method of quantifying the copy number of viral RNA in the sample [7].

According to some studies on the significance of Ct values of PCR in the COVID-19 context, lower Ct values (high viral load) were associated with a worse outcome including prolonged hospitalization of COVID-19 patients, progression to complications as lung fibrosis, and higher mortality rates [8–10]. Other studies reported predictors of mortality in COVID-19 patients to be related to age, sex, chronic diseases, pneumonia, and some laboratory parameters [11]. Patients having one or more predictors of mortality, including those with a high viral load of SARS-CoV-2, are usually located in intensive care units (ICUs) and require extensive medical care and resources. The duration at which patients with high viral load remain at increasing mortality of risk is still undetermined. This study aimed to identify risk factors for prolonged hospitalization and mortality as well as to estimate the temporal point at which the risk of mortality stabilizes among COVID-19 patients with high viral load.

## Methodology

### Study setting

This cross-sectional, single-center, retrospective observational study was carried out in a hospital with a ward for the isolation of COVID-19 patients, in Alexandria, Egypt. The period in which this study occurred was during the 2nd wave of the COVID-19 pandemic (from October 2020 through April 2021). At that time, according to the protocol of the Ministry of Health and Population, the criteria for triaging patients were as follows: PCR-confirmed patients with positive chest imaging, SpO_2_ ≥ 92%, and having risk factor(s) (age above 65–comorbidity–obesity–pregnancy) were considered to have "moderate disease" and were admitted to COVID-19 ward. Patients with SpO_2_ < 92%, PaO_2_/FiO_2_ < 300, respiratory rate > 30 breaths/min, or lung infiltrates > 50% were considered to have “severe disease” and were admitted to intermediate care. Patients with respiratory failure, septic shock, and/or multi-organ dysfunction were admitted to ICUs as they were categorized as patients with "critical illness" [12].

### Sample size calculation

Due to the importance of viral load (indicated by rt-PCR Ct) as a risk factor for COVID-19 severity and mortality, Ct was the selected risk factor for sample size calculation. A minimum required sample size of 250 COVID-19 patients was calculated to determine the association between the cycle threshold values and different clinical and laboratory parameters (80% power). Records of patients who were admitted within 1–2 days of the onset of symptoms were selected in a random sampling technique till reaching the required sample size. We standardized the time of onset of symptoms so that our results on the duration of hospitalization would reflect days since the onset of the disease too. Sample size calculation was based on anticipated findings from a study [13] which determined that the Ct value was correlated with lung disease severity (*r* = − 0.765) and that the average Ct value was lower in patients who died during the study (mean 34.79 ± 2.76) than in those who did not die (mean 37.43 ± 7.62). The calculation was performed using a two-sided Pearson's correlation coefficient as well as an independent sample test at a 0.05 level of significance.

### Data collection

Data of PCR-confirmed COVID-19 patients were collected from hospital and laboratory records. A data sheet was used to record data from patient files, including personal data and medical history. Clinical data of each patient including fever, cough, chest tightness, diarrhea, loss of taste, and smell were recorded. Some admission laboratory test results (complete blood picture, CRP, and rt-PCR Ct values) were obtained from the hospital records. Admission test results of SARS-CoV-2 viral loads from nasopharyngeal swabs were assessed using the TaqMan™ 2019nCoV Assay Kit (Thermo Scientific™) Catalog number: A47532. This kit targets these genes: open reading frame 1ab (ORF 1ab), nucleocapsid (N), and spike (S). According to the manufacturer’s instructions, positive PCR results were those having Ct values > 15−< 37. Results were then categorized into three categories of viral load: Ct values of 25 or less were considered to correspond to “high viral load” while Ct values ranging between 25 and  < 30 were considered to correspond to “moderate viral load” and Ct values of 30– < 37 were considered” low viral load”. We included only Ct results of the admission PCR samples obtained on admission and omitted any subsequent results from the same patient. Chest CTs were categorized according to their CO-RAD scale [6]. The duration of hospitalization (days) was collected from the records as well as the dates of admission and discharge/death (outcome) of patients.

### Ethical consideration

The study protocol and ethical approval for the conduction of this study were granted by the Ethical Committee of the High Institute of Public Health, Alexandria University. The anonymity and confidentiality of patients were assured and maintained.

### Statistical analysis

The data analyses were carried out using R software version 3.6.3 (packages: psych, dplyr, survival, survimir, finalfit, Resource Selection). Continuous variables were expressed as median (interquartile range) after checking the normality of the distribution and the *p*-values were derived using the Kruskal–Wallis test. The categorical variables were expressed as numbers (%), and *p*-values were derived using the Chi-square test or Fisher Exact test in case of violation of the aforementioned test assumptions. A *p*-value < 0.05 (2-tailed) was considered statistically significant throughout the analysis. Univariate and multivariate binary logistic regression models were used to study the predictors of mortality. The results from the model were expressed as odds ratios (95% confidence interval) and a *p*-value for statistical significance. Survival outcome for all patients was also followed up from the date of admission until an arbitrary study end-point date, April 2021, which was used to generate the Kaplan–Meier survival analysis curves and Cox-regression model. The proportional hazard assumption for the Cox-model was examined and graphically presented. The variables included in the model were: CO-RAD, viral load, age, admission lymphocytic count, and sex. We used the STROBE cross-sectional checklist when writing our report [14].

## Results

This study included 519 patients in a private COVID-19 isolation hospital. Almost half of the patients (50.67%) were above 55 years, and those aged 18–35 years were about 39.88%. Patients less than 18 years of age represented only 0.39%. Males contributed 53.95% of the studied patients. The majority of patients (61.46%) were admitted to wards, while 23.89% were in the ICU and 14.65% were in intermediate-care. The most common symptom among all patients was fever (79.38%), followed by cough (78.81%), fatigue (54.9%), and diarrhea (49.9%). Of the total patients, 55.88% were hypertensive, 34.87% were diabetics, and 38.54% were asthmatics. A total of 106 patients (20.42%) were deceased by the end of the study.

Lymphocytic counts ranged between 200 and 7200 cells/cmm (median = 920), and 55.30% of patients were lymphopenic, while only 7.32% had lymphocytosis. The median CRP value was 67 IU/ml. Hospital stay ranged from 5- 40 days (median = 20 days). Concerning the lung CT findings, the CO-RAD scale showed that 42.77% of patients were on a scale of " 5–6", 22.16% were on a scale "of 3", 21.19% were on a scale "of 4" and only 13.87% were on a scale "1–2". The Ct for ORF and N genes was 0–35, while the S gene had a range of 0–34. All three genes had a median of "25". Concerning the Ct value of RT-PCR, 40.08% of patients had a high viral load (low Ct), 42.77% had a moderate viral load, and only 17.15% had a low viral load.

By bivariate analysis, several factors were found to be associated with prolonged hospitalization. These were: older age (*p* < 0.001), diabetes (*p* < 0.001), hypertension (*p* < 0.001), pulmonary embolism (*p* = 0.015), cough (*p* = 0.005), high CRP (*p* < 0.001), high CO-RAD scale (*p* < 0.001), malaise (*p* = 0.017), dyspnea (*p* = 0.007) and high viral load (*p* < 0.001). The longest duration of hospitalization was among those with high viral load (40 days), followed by moderate viral load (35 days), then low viral load (25 days) (Table 1).

**Table 1:** Bivariate analysis of risk factors affecting the duration of hospitalization among the recovered COVID-19 patients

	Length of stay (recovered)		*p*-value
	Median	(Min–max)	
*Age*			
< 18	23	(10–42)	< 0.001*^a^
18–35	20	(10–40)	
35–55	15	(10–35)	
> 55	21	(20–22)	
*Sex*			
Male	22	(10–40)	0.125^b^
Female	20	(10–42)	
*Smoking*			
Yes	22	(10–38)	0.109^b^
No	22	(10–42)	
*Fever*			
Yes	22	(10–42)	0.478^b^
No	22	(10–41)	
*Cough*			
Yes	22	(10–42)	0.005*^b^
No	20	(10–35)	
*Headache*			
Yes	22	(10–42)	0.578^b^
No	22	(10–41)	
*Malaise*			
Yes	21	(10–42)	0.017*^b^
No	22	(10–42)	
*Dyspnea*			
Yes	22	(10–42)	0.007*^b^
No	21	(10–41)	
*Vomiting*			
Yes	22	(10–41)	0.707^b^
No	22	(10–42)	
*Loss of taste and smell*			
Yes	20	(10–40)	0.0631^b^
No	22	(10–42)	
*Hypertension*			
Yes	22	(10–41)	< 0.001*^b^
No	20	(10–42)	
*Diabetes*			
Yes	24	(10–42)	< 0.001*^b^
No	20	(10–38)	
*Asthma*			
Yes	20	(10–41)	0.289^b^
No	22	(10–42)	
*Sore throat*			
Yes	22	(10–42)	0.94^b^
No	22	(10–42)	
*Pulmonary embolism*			
Yes	24	(10–41)	0.015*^b^
No	20	(10–38)	
*Lymphocytic level*			
Lymphopenia	22	(10–42)	< 0.001*^a^
Normal	20	(10–40)	
Lymphocytosis	16	(10–34)	
*C-reactive protein level*			
< 5	17	(10–37)	< 0.001*^a^
5–100	20	(10–40)	
100–200	25	(12–42)	
200–300	30	(10–38)	
> 300	33	(15–41)	
*CO-RAD scale*			
Rad 1–2	14.5	(10–30)	< 0.001*^a^
Rad 3	17	(10–32)	
Rad 4	22	(10–42)	
Rad 5–6	28	(15–41)	
*Viral load*			
High	28	(10–42)	< 0.001*^a^
Intermediate	22	(10–38)	
Low	14	(10–25)	

^a^Kruskal–Wallis test, ^b^Mann–Whitney test

**Fig. 1:**
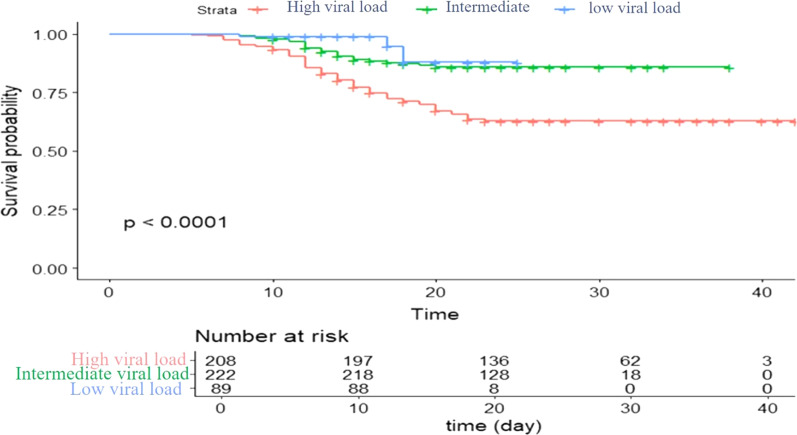
Kaplan–Meier survival plot of the SARS-CoV-2 viral load and the number of patients at risk, cumulative events, and cumulative censoring among COVID-19 patients

Using Kaplan–Meier survival and cumulative mortality analysis (Fig. 1, Additional file 1: Table S1), it was found that the 30 days' cumulative mortality probabilities were as follows: 36.1% (75/208) among patients with high viral load, 12.6% (28/222) for those with moderate viral load, and 3.4% (3/89) among low viral load patients. The log-rank test provided a very significant *p*-value of less than 0.0001, indicating that the difference in survival according to the viral load was highly significant. Among patients with low and moderate viral loads, the cumulative survival for each category of viral loads remained constant with no new deaths beyond "day 20", while mortality among patients with high viral loads stabilized at “day 25”.

**Fig. 2:**
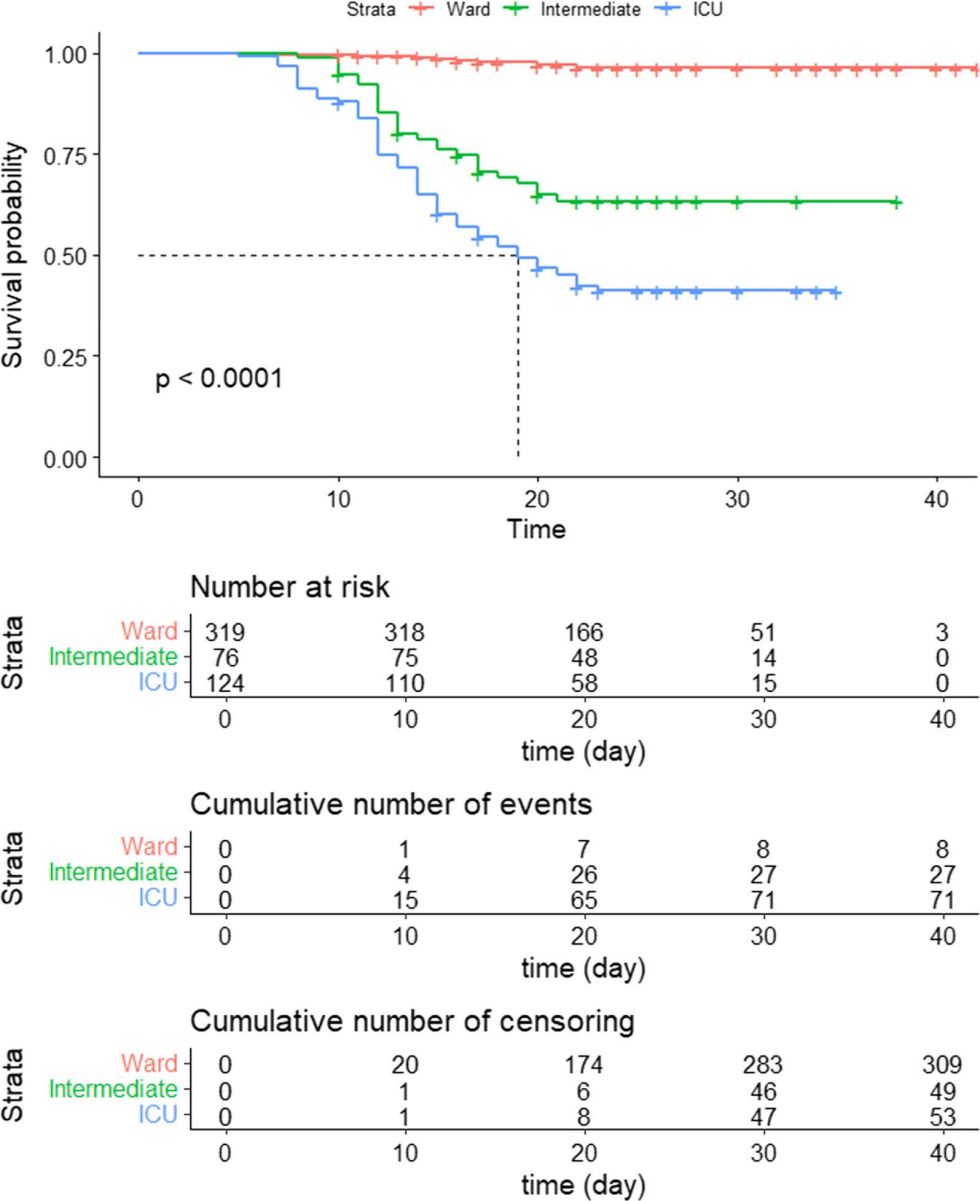
Kaplan–Meier survival plot of the place of admission (ward/ICU/intermediate care) with cumulative risk, cumulative number of events and censoring among COVID-19 patients

The median patient survival was calculated as the time point at which the cumulative survival dropped below 50%. In the ICU-admitted patients, the median survival was 19 days. For patients admitted in wards and the intermediate-care, however, it was not possible to calculate the median patient survival, as in both groups the cumulative survival was more than 50% after 30 days. The mortality rate stabilized at day “25 post-admission" for ICU patients while it stabilized earlier (at day "20") for those admitted to wards and intermediate care. Cumulative mortality rates were 3.7% for patients in wards, 36.6% for those in intermediate care), and 58.9% for ICU patients (Fig. 2 and Additional file 1: Table S2). The log-rank test indicated a highly statistically significant difference between ICU, intermediate-care, and ward-admitted patients (*p* < 0.0001).

According to the logistic regression model (Table 2), when holding all other covariates constant, the adjusted odds ratio (aOR) increased among patients who were: older in age, females, had cough, pulmonary embolism, admitted to intermediate-care or ICU, had higher CO-RAD scale, and/or had lymphocytosis. The presence of “lymphopenia” did not raise the odds of mortality, as opposed to lymphocytosis. Surprisingly, the aOR was inversely proportional to the duration of hospitalization (aOR = 0.17; 95%CI (0.35–0.57). The aOR of “moderate viral load” increased the probability of death by a factor of 2.188 [95%CI (2.001–4.22), *p* = 0.003), which was even higher for patients with high viral load [aOR = 3.024 with 95% CI = 3.01–5.27, *p* < 0.001).

**Table 2:** Logistic regression model with coefficient, *p*-values, odds ratios (95% CI) for mortality among COVID-19 hospitalized patients

Variables	Adjusted odds ratio (aOR)	95%CI of aOR	*p*-value
Intercept	–	–	0.503
Age	1.056	(1.01–3.05)	0.041*
Sex			
Female	1.196	(1.06–11.01)	0.039*
Admission			
Intermediate care	24.026	(11.88–506.08)	< 0.001*
ICU	71.934	(32.51–1912.08)	< 0.001*
Cough	3.372	(2.10–52.02)	0.006*
Pulmonary embolism	8.839	(2.15–624.90)	0.019*
CO-RAD scale	1.527	(2.37–8.25)	< 0.001*
Length of stay	0.170	(0.35–0.57)	< 0.001*
Viral load			
High	3.024	(3.01–5.27)	< 0.001*
Moderate	2.188	(2.001–4.22)	0.003*
Lymphocytic count			
Lymphopenia	0.306	(0.24–2.76)	0.765
Lymphocytosis	3.058	(1.16–58.17)	0.03*

This logistic regression model could predict 99.12% of mortality in our sample, as the pseudo-*R*^2^ (McFadden’s *R*^2^) of the model is 0.9912, *p* < 0.001. The logistic regression equation used was as follows:

[Log (OR death) = 1.174 + 4.18 admission (Post) + 5.28 admission (ICU) + 2.22 cough (yes) + 3.18 Pulmonary embolism (yes) + 1.42 severity of radiation − 0.77 duration of hospitalization + 3.72 viral load (high) + 2.77 viral load (moderate) – 0.18 lymphocytosis + 2.12 lymphopenia + 1.18 sex (female)]. The concordance (c) statistic was identical to the area under the curve (AUC) of the receiver operating characteristic (ROC) curve and the Hosmer and Lemeshow goodness of fit (GOF) test indicated a good fit for the curve (X-squared = 0.31, *df* = 8, *p*-value = 1) (Additional file 1: Fig. S1).

Risk factors identified in logistic regression were also confirmed to be the same in the Cox regression model (Table 3). Older age increased the hazard for death by a factor of 1.02, 95% CI: [1.00–1.03], (*p* = 0.05), holding other variables constant. “Being female” significantly increased the hazard of death by a factor of 1.53; 95% CI: [1.03–2.26], (*p* = 0.031), and the “high CO-RAD scale” increased the hazard of death by a factor of 1.32; 95% CI: [1.06–1.64], (*p* = 0.013). High viral loads significantly increased the hazard of death by a factor of 4.59 (2.38–20.92), *p* = 0.017, while moderate viral load had a lower hazard ratio [2.08 (1.89–3.48), *p* = 0.023]. Although that admission to “intermediate care” had a significant impact on increasing the hazard of death by a factor of 8.25; 95% CI: [3.58–18.98], (*p* < 0.001), this hazard increased by a factor of 15.95; 95% CI: [7.22–35.20], (*p* < 0.001) when the patient was “ICU admitted”, holding all other variables constant. “Lymphocytosis” increased the hazard of death by a factor of 1.89; 95% CI: [1.04–3.45], (*p* = 0.036), while “lymphopenia” did not (Table 3). For this multiple Cox regression model, all coefficients showed statistically significant *p*-values for the Wald test except for weak positivity with “viral load”. Statistically significant *p* values (*p* < 0.001) were found for the following three tests (likelihood ratio, Wald test, and score (log-rank), indicating a significant Cox model. The high concordance value (0.85) indicates a good model performance.

**Table 3:** Univariate and multivariate Cox proportional hazards among COVID-19 hospitalized patients

Variables	Hazard ratio (univariable) 95%CI, *p*-value	Log hazard (β^^^)	Hazard ratio (multivariable) exp(β^^^)95%CI, *p*-value	Probabilistic index
Age	1.04 (1.02–1.06) (*p* < 0.001)*	0.017 (*p* = 0.05)*	1.02 (1.00–1.03) (*p* = 0.05)*	0.496
Sex (female)	1.03 (0.70–1.51) (*p* = 0.890)	0.43 (*p* = 0.03)*	1.53 (1.03–2.26) (*p* = 0.031)*	0.394
CO-RAD	2.05 (1.67–2.51) (*p* < 0.001)*	0.28 (*p* = 0.011)*	1.32 (1.06–1.64) (*p* = 0.013)*	0.423
Viral load				
High load	7.26(2.28–23.11) (*p* < 0.001)*	− 0.53 (*p* = 0.02)*	4.59 (2.38–20.92) (*p* = 0.017)*	0.627
Intermediate load	3.27 (1.04–4.88) (*p* = 0.001)*	− 0.075 (*p* = 0.03)*	2.08 (1.89–3.48) (*p* = 0.023)*	0.501
Type of admission				
Intermediate care	13.74 (6.24–30.26) (*p* < 0.001)*	2.17 (*p* < 0.001)*	8.25 (3.58–18.98) (*p* < 0.001)*	0.108
ICU	26.15 (12.59–54.32) (*p* < 0.001)*	2.84 (*p* < 0.001)*	15.95 (7.22–35.20) (*p* < 0.001)*	0.059
Lymphocytic count				
Lymphopenia	1.09 (0.707–1.67) (*p* = 0.707)	0.31 (*p* = 0.164)	1.36 (0.88–1.04) (*p* = 0.13)	0.423
Lymphocytosis	2.35 (1.31–4.211) (*p* = 0.004)*	0.64 (*p* = 0.04)*	1.89 (1.04–3.45) (*p* = 0.036)*	0.345

There was a statistically significant correlation between high viral load (low Ct values) of the following pairs of genes: ORF and N genes; (*r* = 0.752, *p* < 0.001), N gene and S genes (*r* = 0.687, *p*-value < 0.001) and between the ORF and S genes; (*r* = 0.698, *p* < 0.001). The lymphocytic count was significantly correlated (*p* < 0.001) with Ct values for the 3 genes (inversely correlated with viral load). There was a moderate positive statistically significant correlation also between the duration of hospitalization and the N gene (*r* = 0.688, *p* < 0.001) on the one hand, and the duration of hospitalization and lymphocytic count on the other hand (*r* = − 0.505, *p* < 0.001) (Table 4).

**Table 4:** Correlation matrix between numeric variables using Spearman correlation test

	Age	CRP	Length of stay (days)	Lymphocytic count	Ct of S gene	Ct of ORF gene	Ct of N gene
Age	1						
C-reactive protein	*r* = 0.213^*^ *p*-value < 0.001	1					
Length of stay	*r* = 0.147^*^ *p*-value < 0.001	*r* = 0.277^*^ *p*-value < 0.001	1				
Lymphocytic count	*r* = − 0.130^*^ *p*-value = 0.003	*r* = − 0.505^*^ *p*-value < 0.001	*r* = − 0.218^*^ *p*-value < 0.001	1			
Ct of S gene	*r* = − 0.161^*^ *p*-value < 0.001	*r* = − 0.301^*^ *p*-value < 0.001	*r* = − 0.294^*^ *p*-value < 0.001	*r* = 0.197^*^ *p*-value < 0.001	1		
Ct of ORF gene	*r* = − 0.212^*^ *p*-value < 0.001	*r* = − 0.294^*^ *p*-value < 0.001	*r* = − 0.366^*^ *p*-value < 0.001	*r* = 0.209^*^ *p*-value < 0.001	*r* = 0.698^*^ *p*-value < 0.001	1	
Ct of N gene	*r* = − 0.194^*^ *p*-value < 0.001	*r* = − 0.297^*^ *p*-value < 0.001	*r* = 0.688^*^ *p*-value < 0.001	*r* = 0.239^*^ *p*-value < 0.001	*r* = 0.687^*^ *p*-value < 0.001	*r* = 0.752^*^ *p*-value < 0.001	1

^***^Highly significant correlation, *significant correlation

## Discussion

Approximately 20% of patients with COVID-19 require hospitalization, and a subset of them requires admission to the ICU and remains at a higher risk of mortality for variable periods. The prediction of the duration of hospitalization and the expected patients' stay in the ICU is based on several patients' risk factors. Identification of these risk factors would help in taking proactive measures based on risk stratification of patients. Moreover, it would also help in defining facility-based guidelines and requirements. Defining the point of time after which COVID-19 patients reach a stable risk of mortality is thus important for patient prognosis as well as healthcare facility planning [15, 16].

Viral loads, reflected by Ct values, have been proposed as a surrogate for the calculated viral load and may help in the management of patients [9, 17]. La Scola et al. proposed that the Ct value cut-off of 34 could be used as the level at which patients could be discharged from isolation [3], but Carroll et al., contradicted this recommendation owing to their reported high viral load at this Ct value, indicating high infectivity of patients [18]. In our study, Ct values of 25 or less were considered to correspond to "high viral load" while Ct values ranging between 25 and  < 30 were considered to have "moderate viral load" and those having Ct values of 30– < 37 had" low viral load". In our study, the hospital discharge criteria of patients at the time of this study was to have two consecutive negative RT-PCR results, regardless of the probable earlier clinical resolution of symptoms. This might have contributed to the unnecessary longer periods of hospitalization among some of our patients. These criteria for hospital discharge are no longer applied, as the guidelines changed later during the pandemic, and PCR negativity is no longer a condition for discharge. In our study, lower Ct values (higher viral load) were correlated with prolonged hospitalization as well as being a predictor for mortality. Patients with high viral loads had a risk of death beyond "day 20 post-admission” (their cumulative mortality stabilized at day 25, reaching 36%), in contrast to those with low and moderate viral loads, who reached a lower cumulative mortality rate, at an earlier period. This more delayed peak denotes more aggressive pathology and complications among those with higher viral loads. According to a study by Miller et al., patients with higher Ct values (lower viral loads) had lower odds of mortality (OR for each unit change in Ct value] = 0.94; 95% CI, 0.93–0.96), however, Miller et al., also suggested that no single Ct value cut-off could be clinically recommended for triaging of patients [19].

The stronger correlation between the ORF and N genes than that with the S gene might indicate the relatively less reliability of S gene results in our study; however, more studies from different locations need to confirm our findings. In line with these findings, it was reported that deletion of amino acids 69 and 70 within the spike (S) gene of SARS-CoV-2 B.1.1.7, can result in an undetectable S-gene target (S-gene target failure) in some RT-PCR testing methods [20]. Similarly, a point mutation in the N gene was also linked to N-gene target failure and false-negative PCR results on several Xpert assays [21].

In our study, unfortunately, it was not possible to study the implicated viral variants and relate them to the severity of COVID-19 and its prognosis. This was due to the nature of our study, which was based on hospital records. Testing for SARS-CoV-2 viral variants was not routinely done and thus no data on it was available in our study group. Our study took place in the second wave of the pandemic. During this time, according to other studies in Egypt, the analysis of viral variants sequences using the Pangolin COVID-19 platform showed the presence of 63 lineages, with the predominance of lineages: B, B.1, B.1., B.1.1.1, B.1.1.7 (alpha variant), B.1.170, C.36, and C.36.3 were found to be the predominant lineages. The lineage C.36 continued to circulate till May 2021 and was divided into two other lineages C.36.3 and C.36.3.1 in January 2021 and May 2021 [22, 23].

Variations in viral strains might have an impact on the severity and mortality of COVID-19 in different geographic locations and between pandemic waves. Ong et al. reported that patients infected by B.1.617.2 (delta variant) had higher odds of pneumonia, and severe infection and were more likely to receive remdesivir and/or corticosteroid treatment compared to those infected by B.1.1.7 (alpha variant) and B.1.351 (beta variant) [24]. In another study, the omicron variant was about 75% as likely as a delta variant to cause hospitalization in an unvaccinated person with no history of SARS-CoV-2 infection [25].

In our study, "viral load" was the strongest predictor of mortality among COVID-19 patients (high and moderate viral loads increased the hazard risk of death by 4.59 and 2.08 folds, respectively). Similarly, El Zein et al. reported mortality rates to reach 52% among patients with a high admission viral load, compared to 30% and 16% for patients with moderate and low initial viral loads, respectively [26]. Differences between studies might be attributed to variations in timing and criteria of admission (according to local healthcare guidelines and capacity), and might also be due to population-specific risk factors (age, comorbidities, and genetic causes) as well as the quality of healthcare services provided.

Among patients with low and moderate viral loads, the Kaplan–Meier survival plot demonstrated that the cumulative survival of patients with low and moderate viral loads remained constant with no new deaths beyond "day 20". It was found that on day "25", there was no change in cumulative mortality for patients with high viral load. Although Sousa et al. reported that in their study, survival stabilized on the 24th day of the disease course [27], their study lacked any correlation with viral load. Our study here uniquely reported an earlier stabilization of survival among patients with low and moderate viral load. In our study, although 208 patients had high viral loads, only 124 were admitted to the ICU while the rest were in wards and intermediate care. Due to the associated high risk of mortality among patients with high viral load, it is thus recommended to closely monitor them even when placed in wards, for fear of complications.

The cumulative death rates in the 3 admission locations were highest (58.9%) among the ICU-admitted patients (hazard risk of mortality = 15.95 with 95% CI: [7.22–35.20], *p* < 0.001, OR: 71.93 with 95%CI (32.51–1912.08)] and lowest (3.7%) in the ward-admitted patients. In the ICU-admitted patients, the median survival was 19 days. Elhadi et al. reported much shorter ICU stay among their patients in Libya (7 days among survivors and 6 days among non-survivors) Their shorter ICU stay (compared to ours) was associated with almost similar mortality rates to ours (60.4% and 58.9%, respectively) [28]. One study conducted in several African countries found a mortality rate of 54.7% 30 days after ICU admission, and the authors owed this high rate to limited health resources [29]. Differences in the duration of ICU stay might be related to differences in patient risk factors as well as availability and quality of life supportive measures in the ICU. The association between ICU admission and mortality is expected since these patients were ICU-admitted primarily owing to their worse medical status with higher mortality risk.

According to a study in the USA, mortality was associated with older age, being male, admission to ICU, and having comorbidities (diabetes, hypertension, coronary artery disease, or kidney disease) [19]. Another study from China identified older age, neutrophilia, and organ and coagulation dysfunction as risk factors associated with mortality [30]. In our study, several risk factors contributed significantly to prolonged hospitalization and the risk of mortality. Older age and high CO-RAD scales were risk factors for both parameters, denoting that pathologies associated with older age and higher CO-RAD scales lead to overall worse prognostic interrelated events. Age increased the hazard of death by 1.02 folds [aOR = 1.056 with 95% CI (1.01–3.05)], which was also reported in another study in Egypt on similar patients, where age > 60 years increased the odds of death by 1.3 folds [31]. Older age, with its associated morbidity and weakened immune response, might contribute to higher mortality among such patients. Other proposed mechanisms contributing to increased mortality among the elderly population include low levels of angiotensin-converting enzyme 2 in the elderly, age-dependent difficulty in removing particles from small airways, and excessive release of inflammatory mediators in the elderly [32, 33].

In our study, each increase in the CO-RAD scale increased the hazard of death by 1.32 folds. More aggressive lung involvement in higher CO-RAD scales denotes less functioning lung ability with lower oxygenation, holding the patient at a higher mortality risk as well. Zayed et al. reported that on the CO-RAD scale 4, the sensitivity and specificity for COVID-19 diagnosis were 88% and 98%, respectively [34].

Although females were not at a greater risk for prolonged hospitalization, they had a 1.53-fold increase in the hazard of death. It is often proposed that females are less susceptible to infection than males, possibly because of the protection of the X chromosome and sex hormones, which play an important role in innate and adaptive immunity [35]. Our findings are opposite to that theory and are similar to other studies from India, Nepal, Vietnam, and Slovenia which also reported higher mortality among females [36]. Such differential findings on the association between sex and COVID-19 case fatalities between countries might reflect biases in the case of identification by sex or higher risks for women in certain countries due to demographic factors or countries' health profiles.

Despite that lymphopenia, elevated CRP, diabetes, and hypertension were all significant risk factors for prolonged hospitalization, however, none of them raised the hazard risk for mortality. In contrast, a meta-analysis reported a significant association between elevated CRP levels and mortality rates among COVID-19 patients, despite it is still not clear whether this association is due to a direct effect of the virus in altering biomarker levels, or that abnormal baseline levels predispose a higher individual risk for mortality to COVID-19. Similar to SARS-CoV-2, its earlier ancestor, SARS-CoV-1, was known to cause endothelial dysfunction, leading to an impaired ability to produce nitric oxide and the release of inflammatory markers [37].

The same meta-analysis also reported that hypertension was the comorbidity carrying the highest risk of death among all studied comorbidities (including cardiac, renal, and hepatic diseases and others), conferring a 2.5-fold increase in the odds of death from COVID-19, followed by diabetes which caused a 2.0 fold increase of odds of death in those patients [37]. In our study, unfortunately, data on comorbidities other than hypertension and diabetes were not fully available in all records, as well as other biochemical serum markers, so they were excluded from our statistical analysis.

In contrast to lymphopenia, lymphocytosis raised the odds of mortality [aOR = 3.06 with 95%CI (1.16–58.17)] and hazard risk for mortality by 1.89 folds, despite not being associated with prolonged hospitalization. A study in Egypt also reported that lymphocytosis was a significant predictor of COVID-19 critical illness (they studied severity rather than mortality predictors) [31]. Another study reported that lymphopenia increased the odds of ICU admission by 3.4 folds and the risk of developing acute kidney injury, but no association with mortality was reported [38].

None of the clinical symptoms were found to be significantly associated with a higher hazard risk of mortality, despite that cough, malaise, and dyspnea were associated with prolonged hospitalization (*p* = 0.005, *p* = 0.017, *p* = 0.007, respectively). Zheng et al. reported a significant positive association of dyspnea with COVID-19 progression to severe illness and death and explained that dyspnea suggests poor lung function and decreased oxygenation [35].

COVID-19 pneumonia is associated with a prothrombotic status and increased risk of venous thromboembolic events (deep venous thrombosis and pulmonary embolism). Coagulopathy is a common cause of death in severe COVID-19 patients, and the underlying mechanisms include viral-induced pulmonary endothelial microvascular damage and thrombosis, prolonged immobilization, sepsis, and hypoxia [30]. In our study, there was a significant association between pulmonary embolism and prolonged hospitalization (*p* = 0.015) and higher odds of mortality (aOR: 8.84 with 95%CI (2.15–624.90), which might be a complication of prolonged hospitalization.

### Strengths and limitations

Our report analyzed several factors among COVID-19 patients as being risk factors for prolonged hospitalization and mortality. The Cox regression analysis model was constructed to remove the effects of confounders. Yet, some limitations were present. Data on comorbidities other than hypertension and diabetes were not fully available in all records, so they were excluded from our statistical analysis. The inclusion of more comorbidities might have identified additional predictors of mortality. Similarly, other laboratory parameters might have also been included. Our study was from a single center, and geographical factors might contribute differently in other locations. A correlation between disease severity and outcome with viral variants was not possible.

## Conclusions

High viral load and ICU admission were the two factors with the highest hazard risk of death among COVID-19 patients. Some patients with high viral load are located in the wards and thus should be monitored cautiously for fear of complications. Cumulative mortality stabilizes 5 days earlier among those with low and moderate viral loads than those with high viral loads. Not all factors associated with prolonged hospitalization are necessarily predictors of mortality. Old age, females, high CO-RAD scale, ICU admission, high viral load, and lymphocytosis are significant hazard risks for mortality.

## Supplementary Information

**Additional file 1: Table S1.** Cumulative survival of COVID-19 patients according to their viral load. **Table S2.** Cumulative survival among COVID-19 patients according to location of isolation: wards, intermediate-care, and ICU. **Figure S1.** Receiver Operating Characteristic (ROC) curve to evaluate the discriminative ability of potentially significant predictors.

## Data Availability

The datasets used and/or analyzed during the current study are available from the corresponding author upon reasonable request.

## Abbreviations

ICU: Intensive care unit
COVID-19: Coronavirus Disease 2019
Ct: Cycle threshold
CT: Chest computed topographies
CO-RAD: COVID-19 Reporting and Data System
ORF 1ab: Open reading frame 1ab
RT-qPCR: Reverse transcriptase–quantitative PCR
SARS-CoV-2: Severe acute respiratory syndrome coronavirus 2
S: Spike
N: Nucleocapsid
WHO: World Health Organization
