# Supplementary material for: “Day 25”: a temporal indicator of stabilization of mortality risk among COVID-19 patients with high viral load
Source: Trop Med Health. 2022 Dec 9;50:92. doi: 10.1186/s41182-022-00483-8 (PMC9732988; doi:10.1186/s41182-022-00483-8)
Supplement: Supplementary file 1 — Additional file 1: Table S1. Cumulative survival of COVID-19 patients according to their viral load. Table S2. Cumulative survival among COVID-19 patients according to location of isolation: wards, intermediate-care, and ICU. Figure S1. Receiver Operating Characteristic (ROC) curve to evaluate the discriminative ability of potentially significant predictors. [file 41182_2022_483_MOESM1_ESM.docx]

**Table S1: Cumulative survival of COVID-19 patients according to their viral load**

| Type of viral load | Time (day) | no. at risk | no. of event | Cumulative survival^1^ | Cumulative mortality (1- survival) | 95%CI |
| --- | --- | --- | --- | --- | --- | --- |
| High load  N=208 | 0 | 208 | 0 | 1.000 | 0.00 | (1.00-1.00) |
|  | 5 | 208 | 1 | 0.995 | 0.005 | (0.986- 1.00) |
|  | 10 | 197 | 13 | 0.933 | 0.067 | (0.899-0.967) |
|  | 15 | 163 | 33 | 0.773 | 0.227 | (0.717-0.832) |
|  | 20 | 136 | 20 | 0.671 | 0.329 | (0.610- 0.739) |
|  | 25 | 92 | 8 | 0.628 | 0.372 | (0.564 -0.699) |
|  | 30 | 62 | 0 | 0.628 | 0.372 | (0.564-0.699) |
|  | 35-40 | 16 | 0 | 0.628 | 0.372 | (0.564-0.699) |
| Moderate load  N=222 | 0 | 222 | 0 | 1.000 | 0.00 | (1.00-1.00) |
|  | 5 | 222 | 0 | 1.000 | 0.00 | (1.00-1.00) |
|  | 10 | 218 | 5 | 0.977 | 0.023 | (0.958 -0.997) |
|  | 15 | 171 | 18 | 0.890 | 0.110 | (0.848-0.933) |
|  | 20 | 128 | 5 | 0.859 | 0.141 | (0.811-0.909) |
|  | 25 | 48 | 0 | 0.859 | 0.141 | (0.811-0.909) |
|  | 30 | 18 | 0 | 0.859 | 0.141 | (0.811-0.909) |
|  | 35 | 1 | 0 | 0.859 | 0.141 | (0.811-0.909) |
| Low load  N=89 | 0 | 89 | 0 | 1.000 | 0.00 | (1.00-1.00) |
|  | 5 | 89 | 0 | 1.000 | 0.00 | (1.00-1.00) |
|  | 10 | 88 | 1 | 0.989 | 0.011 | (0.967-1.00) |
|  | 15 | 44 | 0 | 0.989 | 0.011 | (0.967-1.00) |
|  | 20 | 8 | 2 | 0.880 | 0.120 | (0.743-1.00) |
|  | 25 | 2 | 0 | 0.880 | 0.120 | (0.743-1.00) |

*^1^ Cumulative survival is calculated as the proportion surviving on this day/ cumulative survival over the previous period.*

**Table S2: Cumulative survival among COVID-19 patients according to location of isolation: wards, intermediate-care, and ICU**

| Location of admission | Time (days) | no. at risk | no. of events | Cumulative survival | Cumulative mortality (1- survival) | 95%CI |
| --- | --- | --- | --- | --- | --- | --- |
| Ward  N=319 | 0 | 319 | 0 | 1.000 | 0 | (1.00 -1.00) |
|  | 5 | 319 | 0 | 1.000 | 0 | (1.00 -1.00) |
|  | 10 | 318 | 1 | 0.997 | 0.003 | (0.991-1.00) |
|  | 15 | 240 | 3 | 0.986 | 0.014 | (0.972-1.00) |
|  | 20 | 166 | 3 | 0.970 | 0.030 | (0.949-0.993) |
|  | 25 | 83 | 1 | 0.963 | 0.037 | (0.938-0.990) |
|  | 30 | 197 | 0 | 0.963 | 0.037 | (0.938-0.990) |
|  | 35 | 193 | 0 | 0.963 | 0.037 | (0.938-0.990) |
| Intermediate- care  N=76 | 0 | 76 | 0 | 1.000 | 0 | (1.00 -1.00) |
|  | 5 | 76 | 0 | 1.000 | 0 | (1.00 -1.00) |
|  | 10 | 75 | 4 | 0.947 | 0.053 | (0.898-0.999) |
|  | 15 | 58 | 14 | 0.760 | 0.240 | (0.669-0.863) |
|  | 20 | 48 | 8 | 0.648 | 0.352 | (0.548-0.767) |
|  | 25 | 28 | 1 | 0.634 | 0.366 | (0.532-0.754) |
|  | 30 | 14 | 0 | 0.634 | 0.366 | (0.532-0.754) |
|  | 35 | 2 | 0 | 0.634 | 0.366 | (0.532-0.754) |
| ICU  N=124 | 0 | 124 | 0 | 1.00 | 0 | (1.00 -1.00) |
|  | 5 | 124 | 1 | 0.992 | 0.008 | (0.976-1.000) |
|  | 10 | 110 | 14 | 0.879 | 0.121 | (0.823-0.938) |
|  | 15 | 80 | 34 | 0.602 | 0.398 | (0.522-0.695) |
|  | 20 | 58 | 16 | 0.468 | 0.532 | (0.387-0.566) |
|  | 25 | 31 | 6 | 0.411 | 0.589 | (0.331-0.510) |
|  | 30 | 15 | 0 | 0.411 | 0.589 | (0.331-0.510) |
|  | 35 | 2 | 0 | 0.411 | 0.589 | (0.331-0.510) |

*^1^ Cumulative survival is calculated as the proportion surviving on this day! cumulative survival over the previous period.*


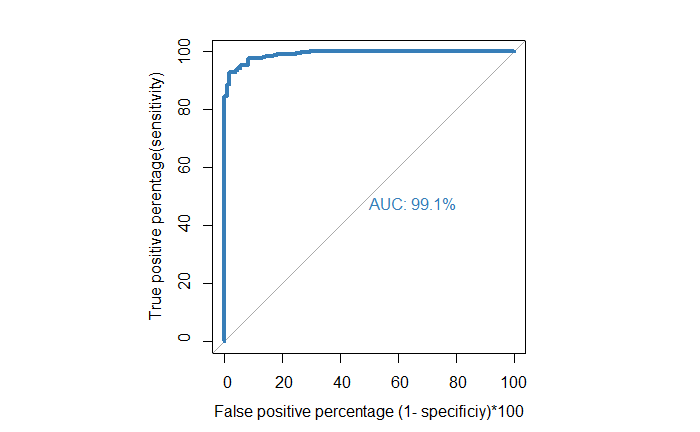


**Figure S1: Receiver Operating Characteristic (ROC) curve to evaluate the discriminative ability of potentially significant predictors**
